# Predictors of New and Persistent New Left Bundle Branch Block One Year after the Implantation of a Sutureless and Rapid-Deployment Aortic Valve Prosthesis

**DOI:** 10.3390/diseases11030100

**Published:** 2023-08-03

**Authors:** Rafał Januszek, Robert Balan

**Affiliations:** 1 Department of Cardiology and Cardiovascular Interventions, University Hospital, 30-688 Kraków, Poland; 2 Department of Cardiac Surgery, Klinikum Passau, 94-032 Passau, Germany; balanrobert2003@gmail.com

**Keywords:** atrio-ventricular conduction abnormalities, left bundle branch block, risk factors, sutureless and rapid-deployment aortic valve

## Abstract

Introduction: Conduction disorders following aortic valve replacement therapy (AVR), either surgical or percutaneous, are related to a higher risk of complete atrioventricular block and permanent pacemaker implantation (PPI). Aim: The objective of this study was to assess risk factors regarding the incidence of new postoperative and persistent new left bundle branch block (LBBB) 1 year after the implantation of a sutureless/rapid-deployment (SURD) aortic valve prosthesis. Material and Methods: The current study included 200 consecutive patients treated with isolated or concomitant AVR between May 2014 and May 2017 at the Department of Cardiac Surgery in Pasawa with SURD aortic valve EDWARDS INTUITY Elite^TM^ implantation. The patients were divided according to the presence of new postoperative LBBB (67 patients, 33.5%) and persistent new LBBB 1 year after AVR (35 patients, 17.5%). A comparative analysis was performed between patients with and without new LBBB after AVR and those with and without persistent LBBB 1 year after AVR. Univariate and multivariate regression analyses were conducted to extract the risk factors of LBBB occurrence. Results: Among the risk factors for the lack of new LBBB development after AVR, Euroscore II (*p* < 0.001) was found, while for the occurrence of persistent new LBBB 1 year after AVR, atrial fibrillation (*p* = 0.001), length of hospital stay (*p* = 0.001) and body mass index (*p* = 0.004) were noted. Conclusions: Patients with new or persistent new LBBB 1 year after AVR had lower mean Euroscore II and BMI values. Their stay at the hospital was also shorter.

## 1. Introduction

Patients undergoing conventional aortic valve replacement (AVR) are related to an increased risk of atrioventricular conduction disorders [1]. One of the most serious consequences of these conduction disturbances is the development of advanced or complete atrioventricular blocks associated with permanent pacemaker implantation (PPI) [2]. The incidence of atrioventricular conduction disturbances is related to the percutaneous treatment method and is higher with transcatheter aortic valve implantation (TAVI) compared to standard surgery [3]. It seems that the degree of decalcification and the technique of valve implantation is of major importance here. In the case of TAVI procedures, decalcification is, by definition, not possible. This is due to the pressure of the pressed calcifications on the heart’s conductive structures. In selected populations, following TAVI procedures using the CoreValve prosthesis, PMI was even reported to reach 36% [4]. Also, the technique of valve deployment during TAVI is related to the frequency of PPI and was found to be greater in the group of early-generation self-expanding compared to balloon-expandable valves [5]. It has been demonstrated that the implantation of a sutureless/rapid-deployment (SURD) aortic valve prosthesis is related to higher PPI rate in comparison to a stent-sutured bioprosthesis (SAVR) [6]. Furthermore, the frequency of intraventricular conduction abnormalities, such as left (LBBB) and right bundle branch blocks (RBBB), is increased with prosthetic aortic valve implantation. In previously published studies, it has been demonstrated that the occurrence of LBBB after surgical AVR was 15.6% [7], while the combined rate of LBBB and RBBB was 9.2% [8]. The incidence of new LBBB after TAVI was reported to be 25% [9]. In another publication by Khounlaboud et al., the authors reported new LBBB occurrence in 4.6% of patients after SAVR compared to 16.4% of patients treated with TAVI during the study period [10]. It has been shown that after almost 2 years of follow-up, new-onset LBBB was not associated with increased mortality [11]. Higher pacemaker implantation rates were observed in patients with new-onset LBBB after SURD [11]. In another meta-analysis, better outcomes were indicated regarding the frequency of PPI after rapid-deployment AVR when compared to sutureless AVR [12]. While analyses of PPI risk factors after AVR have been published, predictors of LBBB remain poorly available [13].

Therefore, in the current study, we aimed to assess risk factors regarding the incidence of new postoperative and persistent new left bundle branch block (LBBB) 1 year after the implantation of a sutureless/rapid-deployment (SURD) aortic valve prosthesis.

## 2. Methods

### 2.1. Patients

Between May 2014 and May 2017, 200 consecutive patients underwent surgical AVR via the EDWARDS INTUITY Elite SURD prosthesis, which required obtaining a certificate. More detailed patient characteristics have been presented in our prior publication [14]. The prosthesis type remained at the discretion of the first operator. All patients were included in the trial and their files screened for the occurrence of new postoperative conduction abnormalities. These included: LBBB, RBBB, atrioventricular (AV) block III and PMI. ECGs were recorded at the intensive care unit (ICU) every following day, the day after transfer to the regular ward, and 1 day prior to discharge unless otherwise stated. The occurrence of new conduction abnormalities was evaluated on the basis of the final electrocardiogram carried out 1 day before discharge. We considered LBBB 1 year after surgery to be consistent in only those patients who had persistent block from the time of onset, which occurred in the period between AVR and the final assessment before discharge from the hospital. This group did not include patients who developed LBBB during the period between hospital discharge and the assessment 1 year after discharge. The usual pre-, peri- and postoperative indicators retrieved from the patients’ files were also considered.

### 2.2. Surgical Techniques 

Isolated AVR, concomitant procedures and isolated aortic valve procedures (both minimally invasive—ministernotomy in the 3rd or 4th intercostal space and full sternotomy procedures) were included. More detailed characteristics of surgical techniques have been described in our previous publication [14]. 

### 2.3. Technique for Implanting the SURD EDWARDS INTUITY Valve

The rapid-deployment valve was implanted as recommended by Edwards Lifesciences, Irvine, CA, USA. All 5 surgeons were certified by the company. Both conventional AVR surgical procedures and aortic annus decalcification did not differ. After decalcification and rinsing, the annulus was measured (Edwards Lifesciences barrel sizer and the replica). When the barrel fitted the annulus without applying force or sliding into the left ventricle, correct size was assumed. Other detailed characteristics of the procedure have been given in a previous publication [14]. 

### 2.4. Anaesthetic and Intensive Care Treatment

In all patients, anesthesia was induced and maintained according to the previously described methods [14]. 

### 2.5. Data Acquisition and Statistical Analysis

The complete clinical data were extracted from patient documentation. Categorical variables are given as numbers as well as percentages and compared using Pearson’s chi-squared or Fisher’s exact tests. This was performed when 20% of the cell count was below 5.

The continuous variables are demonstrated as means ± standard deviations. The Mann–Whitney U test was applied for variables lacking normal distribution. This was also performed when 20% of the cells had a count below 5.

In the case of non-normal distribution, variables are given as medians and interquartile ranges. The equality of variance was tested using Levene’s test, while intergroup differences were compared via the Student’s or Welch’s *t*-tests. This was dependent on the equality of variance regarding normally distributed variables. 

The ordinal variables were further compared via the Cochran–Armitage trend test. All baseline, demographic and procedural characteristics were assumed as possible predictors of new LBBB after AVR and persistent new LBBB 1 year after AVR in univariate linear mixed effect models with the clustering effect of multiple stents/procedures per single patients treated as random effects. Then, variables with a *p*-value < 0.2 or those of supposed significance were added to the multivariate model. Final multivariate regression models were designed by minimizing Akaike information. The criteria were applied to discover predictors of stent expansion in the U and non-U software groups. All performed statistical analyses were carried out using JMP^®^, Version 13.1.0 (SAS Institute INC., Cary, NC, USA) and R 4.1.1 (R Foundation for Statistical Computing, Vienna, Austria, 2021) with the “lme4” package, version 1.1-27.1.

## 3. Results

In the current analysis, 67 (33.5%of the overall group) patients presented new LBBB after the AVR procedure. One year after AVR, 35 (18.2%of the overall group and 52.2% of those who initially presented new LBBB after surgery) patients demonstrated persistent LBBB among those who had survived. Therefore, it can be concluded that in 32 (47.7%) patients, LBBB resolved during the 1-year follow-up.

### 3.1. Baseline Clinical Characteristics, Echocardiography and Electrocardiography before Aortic Valve Replacement Surgery

Patients with new LBBB after AVR (*p* < 0.001) and persistent new LBBB 1 year after AVR (*p* = 0.009) were related to a significantly lower mean Euroscore II value when compared to patients without any new occurrences of LBBB. Patients without new LBBB were more often affected by endocarditis before surgery (*p* = 0.03). Also, the mean left ventricular ejection fraction was greater in patients with new LBBB after AVR in comparison to those without its implementation (*p* = 0.04) (Table 1).

### 3.2. Procedural Indices 

REDO cardiac surgery occurred more frequently in the group of patients without new LBBB incidence after AVR in contrast to those who presented LBBB (*p* = 0.007). Moreover, ministernotomy was more often observed in patients with new LBBB after AVR in comparison to those without its introduction (*p* = 0.015). Considering surgery duration, cardiopulmonary bypass (*p* = 0.05) and operation time (*p* = 0.01) were shorter among the group of patients with persistent LBBB 1 year after AVR compared to those without it (Table 2).

### 3.3. Procedural Complications

The length of hospital stay was significantly longer in patients without new LBBB after AVR (*p* = 0.002) and persistent new LBBB 1 year after AVR (*p* = 0.03). The new RBBB occurrence (*p* = 0.001) and pacemaker implantation (*p* = 0.02) after AVR were found more often in the group of patients without new LBBB after AVR in comparison to the group of patients with new LBBB after AVR (Table 3).

### 3.4. One Year Follow-Up

Sinus heart rhythm was observed more frequently in the group of patients with new LBBB after AVR (*p* < 0.001) and persistent new LBBB 1 year after AVR (*p* < 0.001). Other selected indices at the 1-year follow-up are presented in Table 4. 

### 3.5. Predictors of New LBBB after AVR and Persistent New LBBB 1 Year after AVR—Univariate Analysis

Among the significant risk factors of lower new LBBB occurrence assessed via univariate analysis after AVR, the following were found: lower Euroscore II value, no REDO operation, partial sternotomy, no pacemaker implantation post-AVR and shorter length of hospital stay. Meanwhile, among the risk factors of greater persistent new LBBB incidence after AVR, lower body mass index, lower Euroscore II value, no REDO operation, shorter length of operation and shorter length of hospital stay were noted (Table 5). 

### 3.6. Predictors of New LBBB after AVR and Persistent New LBBB 1 Year after AVR—Multivariate Regression Analysis

**Figure 1:**
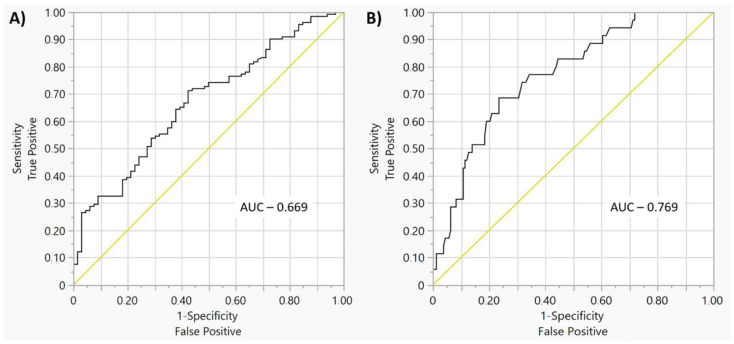
(**A**). Receiver operating characteristic *curve for the model of the relationship between selected indices (explained in the description of statistical methods) and new LBBB appearance after AVR procedure—multivariate analysis*. (**B**). Receiver operating characteristic *curve for the model of the relationship between selected indices (explained in the description of statistical methods) and persistent new LBBB following 1 year of follow-up after AVR procedure—multivariate analysis. AUC: area under the curve*.

Among the significant risk factors of new LBBB occurrence assessed via multivariate regression analysis after AVR, it was found that lower mean Euroscore II value was related to greater new LBBB incidence after AVR (OR: 1.202, 95% CI: 1.063–1.404; *p* = 0.001). Among the risk factors regarding the incidence of persistent new LBBB after AVR, the following were observed: atrial fibrillation (OR: 17.622, 95% CI: 3.029–152.422; *p* = 0.001), shorter length of hospital stay (OR: 0.896, 95% CI: 0.806–0.969; *p* = 0.004) and lower mean body mass index before procedure (OR: 0.873, 95% CI: 0.788–0.959; *p* = 0.004) (Table 6 and Figure 1).

## 4. Discussion

The main findings of the current publication were first that patients with new LBBB after AVR and persistent new LBBB 1 year after AVR were related to significantly lowe mean Euroscore II value when compared to patients without new LBBB. Also, endocarditis occurred in more of the patients without new LBBB after AVR in comparison to the group of patients with new LBBB. The mean left ventricular ejection fraction was greater in patients with new LBBB after AVR. Secondly, REDO cardiac surgery occurred more frequently, while ministernotomy was less frequent in the group of patients without new LBBB incidence after AVR in comparison to those who presented with LBBB. The length of operation and cardiopulmonary bypass time were shorter among the group of patients with persistent LBBB 1 year after AVR. Thirdly, the length of hospital stay was significantly longer in patients without new and persistent new LBBB 1 year after AVR. Fourthly, among the significant risk factors of new LBBB occurrence assessed via multivariate regression analysis after AVR, lower Euroscore II was found, while risk factors concerning the incidence of persistent new LBBB after AVR included atrial fibrillation, the length of hospital stay and body mass index. 

The first observation that emerges from the analysis of the results is that patients with a lower cardiac load, higher left ventricular ejection fraction and leaner prognosis in terms of lower LBBB incidence in the postoperative period and follow-up demonstrate less persistent LBBB after the 1-year follow-up. This was also associated with shorter surgery time and duration of hospitalization. The length of hospitalization is closely related to the lower incidence of endocarditis in patients without new and persistent LBBB 1 year after AVR. The length of hospitalization was also closely connected with the higher incidence of partial sternotomy and lower REDO surgery in the group of patients with new or persistent LBBB 1 year after AVR.

Considering the frequency of LBBB occurrence after surgical AVR, the frequency of new LBBB following AVR seems to be greater than in other studies comparing surgical patients, including the most recently published analysis comprising patients after SURD AVR, in which the frequency reached more than 17% [8]. In a study carried out by D’Onofrio et al., it was revealed that nearly 40% of patients develop a new conduction disorder after rapid-deployment aortic valve implantation [15]. Of these, 1/3 recover after 1 year. Bioprosthesis size and age were identified as independent risk factors for the occurrence of conduction disorders after surgery [14]. In another study, it was also demonstrated that sutureless AVR is associated with an increased risk of new-onset LBBB and PPI requirement compared to conventional AVR. In this study, similarly to those presented by our team, it was confirmed that postoperative conduction disorders do not affect mid-term survival rate [16]. In the research conducted by Reggeer et al., it has been shown that sutureless surgical AVR and TAVI procedures were related to a higher incidence of new-onset LBBB at hospital discharge (23% and 16%, respectively) when compared to patients treated with conventional AVR (4%; *p* < 0.001). This was further confirmed by multivariate logistic regression analysis [17]. The frequency of new LBBB after TAVI was reported to be significantly greater in comparison to surgical AVR and dependent on the method. For self-expandable valves, the value was reported to be greater, between 29 and 65% [18,19,20,21,22,23,24,25], while for balloon-expandable valves, the value was lower, between 12 and 18% [20,24,26,27,28]. In patients undergoing TAVI, LBBB occurrence was significantly related to a more frequent complete atrioventricular block as well as PPI, and not with mortality [26]. In our analysis, there were no noted significant relationships between the abovementioned features. Among the factors found to be significantly correlated with the occurrence of LBBB in patients treated with TAVI, Poels et al. found the effective distance between the aortic valve and conduction system [8]. This relationship is likely reflected in our analysis, because in leaner patients, LBBB occurred more frequently after AVR, and leaner patients were more expected to have a shorter distance between the aortic valve and conduction system. In such estimates, the annulus diameter of the implanted valve and the ratio of the distance between the valve and the conducting system as well as the diameter of the implanted valve may also have potential influence. Among other risk factors of LBBB development after TAVI, the presence of a bicuspid aortic valve was demonstrated over the tricuspid one [29]. In our analysis, all patients had tricuspid valves; thus, we were not able to analyze that point. In earlier studies, it has been suggested that a new BBB acquired after AVR is associated with an increased adverse event rate. However, this relationship was not confirmed in our analysis or in several other trials [7,16]. Based on the results of the research under analysis, it seems that healthier patients, less burdened with concomitant diseases, tolerated AVR less well in terms of higher LBBB incidence, as did patients with higher left ventricular ejection fraction. On the other hand, it may also be concluded that sicker patients (those with greater Euroscore value and more burdened with other concomitant diseases) developed more severe conductivity complications, such as third-degree AV block and subsequent pacemaker implantation. For example, patients with endocarditis were likely to develop more advanced atrioventricular conduction disorders. A similar relationship was observed for CPB time and length of hospitalization, which suggests that healthier patients developed less serious complications related to atrioventricular conduction more frequently, while those who were sicker often required additional interventions, such as pacemaker implantation. However, in the case of REDO operations, it can be concluded that hospital stays are longer, and this would not be related to the above conclusions. Nonetheless, it is not a rule that REDO operations are longer. To the contrary, in many cases, they are even shorter if no complications occur.

## 5. Conclusions

Patients with new LBBB after AVR and persistent new LBBB 1 year after AVR did not differ in the frequency of PPI implantation from the non-LBBB population. Patients with new or persistent new LBBB 1 year after AVR were healthier, presented atrial fibrillation more often, were slimmer and stayed in the hospital for a shorter period time.

## 6. Limitations

One of the main limitations of the study is its small sample size. The occurrence of new conduction abnormalities was assessed based on the final electrocardiogram 1 day before discharge. Therefore, some readers may consider the results underestimated due to the possibility of postoperative conduction disorders that subsided before discharge from the hospital. However, we considered such conduction disturbances in the postoperative period as coexisting with the procedure itself. It should also be emphasized that some patients had a pacemaker or LBBB at baseline, and it is not possible to demonstrate whether these patients developed new intraventricular conduction disorders after the procedure, especially in patients with a pacemaker. This fact could have influenced the calculations concerning the predictors of the occurrence of new intraventricular conduction disorders after the procedure. However, on the other hand, removing these patients would create a new, artificial population that does not exist in the real world, distorting the estimated incidence of conduction disturbances at baseline before surgery in this group of patients.

## Figures and Tables

**Table 1:** Clinical characteristics and electrocardiography before aortic valve replacement according to the presence of LBBB directly post-surgery and after 1 year of follow-up.

	Total	New LBBB after AVR			Persistent New LBBB 1 Year after AVR		
		No (n = 133)	Yes (n = 67)	*p*-Value	No (n = 157)	Yes (n = 35)	*p*-Value
**Group 1 vs. 2**	100 (50%)	66 (49.6)	34 (50.7)	0.88	81 (51.6)	16 (45.7)	0.52
Age, years	71.3 ± 7.5 72.5 (67; 77)	71.8 ± 7.7 74 (68; 77)	70.1 ± 7.2 72 (64; 76)	0.08	71.1 ± 7.8 72 (67; 77)	70.8 ± 6.5 72 (65; 76)	0.52
Gender, male	118 (59)	80 (60.1)	38 (56.7)	0.64	95 (60.5)	20 (57.1)	0.71
Body mass index, kg/m^2^	28.3 ± 4.4 28 (25; 31)	28.6 ± 4.03 28 (25; 31)	27.6 ± 5.2 27 (24; 31)	0.15	28.6 ± 4.3 28 (25; 31.5)	26.9 ± 4.9 26 (23; 31)	0.06
Euroscore II, %	3.8 ± 4.3 2.3 (1.3; 4.2)	4.4 ± 5 2.5 (1.7; 4.8)	2.5 ± 2 1.9 (1; 3.6)	<0.001	3.9 ± 4.4 2.4 (1.4; 4.3)	2.4 ± 2 1.4 (1; 3.4)	0.009
New York Heart Association (NYHA) class grade							
I	1 (0.5)	1 (0.7)	0 (0)	0.38	1 (0.6)	0 (0)	0.13
II	19 (9.5)	12 (9)	7 (10.4)		13 (8.3)	6 (17.1)	
II/III	1 (0.5)	0 (0)	1 (1.5)		0 (0)	1 (2.9)	
III	160 (80)	105 (78.9)	55 (82.1)		128 (81.5)	26 (74.3)	
IV	19 (9.5)	15 (11.3)	4 (6)		15 (9.5)	2 (5.7)	
LVEF, %	54.6 ± 9.7 60 (50; 60)	53.7 ± 10.3 60 (50; 60)	56.3 ± 8.2 60 (60; 60)	0.04	54.5 ± 9.9 60 (50; 60)	56.2 ± 7.6 60 (60; 60)	0.31
Endocarditis (indication for AVR)	9 (4.5)	9 (6.8)	0 (0)	0.03	7 (4.5)	0 (0)	0.35
Arterial hypertension	186 (93)	126 (94.7)	60 (89.5)	0.23	147 (93.6)	31 (88.6)	0.29
Diabetes mellitus	66 (33)	47 (35.3)	19 (28.4)	0.32	52 (33.1)	13 (37.1)	0.64
Prior stroke	29 (14.5)	20 (15)	9 (13.4)	0.76	22 (14)	5 (14.3)	1.0
Dialysotherapy	5 (2.5)	4 (3)	1 (1.5)	0.66	4 (2.5)	1 (2.9)	1.0
Atrioventricular conduction and heart rhythm disturbances before AVR							
Sinus rhythm	166 (83)	106 (79.7)	60 (89.5)	0.08	130 (82.8)	32 (91.4)	0.2
Atrial fibrillation	28 (14)	21 (15.8)	7 (10.4)	0.3	22 (14)	3 (8.6)	0.58
Pacemaker	5 (2.5)	5 (3.8)	0 (0)	0.17	4 (2.5)	0 (0)	1.0
AICD	4 (2)	3 (2.3)	1 (1.5)	1.0	4 (2.5)	0 (0)	1.0
RBBB	16 (8)	16 (12)	0 (0)	0.003	15 (9.5)	0 (0)	0.07
LBBB	11 (5.5)	11 (8.3)	0 (0)	0.017	9 (5.7)	1 (2.9)	0.69
LAHB	5 (2.5)	5 (3.8)	0 (0)	0.17	5 (3.2)	0 (0)	0.58

Data are expressed as mean ± standard deviation and median, interquartile range or numbers (percentages). AICD: automated implantable cardioverter defibrillator; AVR: aortric valve replacement; LAHB: left anterior hemiblock; LBBB: left bundle branch block; LVEF: left ventricle ejection fraction; RBBB: *right bundle branch block*. Group 1: first 100 cases of patients in a row who underwent surgical AVR using the EDWARDS INTUITY Elite rapid-deployment aortic valve prosthesis. Group 2: second 100 cases of patients in a row who underwent surgical AVR using the EDWARDS INTUITY Elite rapid-deployment aortic valve prosthesis.

**Table 2:** Procedural indices according to the presence of LBBB directly post-INTUITY Elite rapid valve deployment and after 1 year of follow-up.

	Total	New LBBB after AVR			Persistent New LBBB 1 Year after AVR		
		No (n = 133)	Yes (n = 67)	*p*-Value	No (n = 157)	Yes (n = 35)	*p*-Value
EDWARDS INTUITY Elite Rapid-Deployment Aortic Valve Prosthesis Size							
19	8 (4)	6 (4.5)	2 (3)	0.62	6 (3.8)	1 (2.9)	0.48
21	35 (17.5)	25 (18.8)	10 (14.9)		28 (17.8)	5 (14.3)	
23	63 (31.5)	42 (31.6)	21 (31.3)		53 (33.8)	9 (25.7)	
25	75 (37.5)	45 (33.8)	30 (44.8)		53 (33.8)	18 (51.4)	
27	19 (9.5)	15 (11.3)	4 (6)		17 (10.8)	2 (5.7)	
INTUITY second valve size, if required							
23	1 (100)	1 (100)	-	-	1 (100)	-	-
Magna size, if required							
21 25	2 (66.7) 1 (33.3)	2 (100) 0 (0)	0 (0) 1 (100)	0.33	2 (66.7) 1 (33.3)	- -	- -
Tricuspid valve replacement	2 (1)	1 (0.7)	1 (1.5)	1.0	2 (1.3)	0 (0)	1.0
Mitral valve replacement	5 (2.5)	5 (3.8)	0 (0)	0.17	3 (1.9)	0 (0)	1.0
Coronary artery bypass grafting	65 (32.5)	23 (34.3)	42 (31.6)	0.69	51 (32.5)	11 (31.4)	0.9
Ascending aorta replacement	11 (5.5)	9 (6.8)	2 (3)	0.34	9 (5.7)	1 (2.9)	0.69
Pulmonary vein ablation	14 (7)	11 (8.3)	3 (4.5)	0.39	13 (8.3)	1 (2.9)	0.47
Left atrial appendage clip	11 (5.5)	10 (7.5)	1 (1.5)	0.1	10 (6.4)	1 (2.9)	0.69
REDO cardiac surgery	23 (11.5)	21 (15.8)	2 (3)	0.007	21 (13.4)	1 (2.9)	0.08
Myectomy	1 (0.5)	1 (0.7)	0 (0)	1.0	1 (0.6)	0 (0)	1.0
Surgeon *							
1 2 3 4 5	104 (52) 58 (29) 4 (2) 27 (13.5) 7 (3.5)	64 (48.1) 40 (30.1) 4 (3) 22 (16.5) 3 (2.3)	40 (59.7) 18 (26.9) 0 (0) 5 (7.5) 4 (6)	0.09	83 (52.9) 45 (28.7) 0 (0) 3 (8.6) 4 (11.4)	18 (51.4) 10 (28.6) 4 (2) 27 (13.5) 7 (3.5)	0.07
Partial sternotomy	64 (32)	35 (26.3)	29 (43.3)	0.015	51 (32.5)	13 (37.1)	0.59
Clamp time, minutes	74.8 ± 31.3 66.5 (53; 84.7)	75.6 ± 30.9 69 (53.5; 86)	73 ± 32.3 60 (53; 83)	0.27	74.2 ± 30.5 68 (53; 84.5)	69.1 ± 29 60 (52; 70)	0.14
Cardiopulmonary bypass time, minutes	111.3 ± 46.2 97 (82; 126)	113.6 ± 46.3 102 (84; 129)	106.5 ± 45.8 90 (79; 115)	0.17	110.6 ± 43.1 99 (83; 126)	98.9 ± 38.3 87 (77; 105)	0.05
Duration of surgery, minutes	206.8 ± 67.6 186 (160; 238)	210.6 ± 67.9 188 (164; 240)	199.4 ± 66.7 180 (156; 220)	0.16	208 ± 65.5 190 (164; 239)	182.3 ± 51.7 175 (146; 195)	0.01

Data are expressed as mean ± standard deviation and median; interquartile range or numbers (percentages). *: numbers 1–5 correspond to the following surgeon performing the SURD procedure.

**Table 3:** Procedural complications after aortic valve replacement during hospitalization.

	Total	New LBBB after AVR			Persistent New LBBB 1 Year after AVR		
		No (n = 133)	Yes (n = 67)	*p*-Value	No (n = 157)	Yes (n = 35)	*p*-Value
Duration of hospital stay, days	17.5 ± 12.2 14 (11.7; 18)	18.8 ± 12.7 15 (12; 21)	15 ± 10.7 13 (10; 15)	0.002	18.5 ± 13.3 14 (12; 19)	13.4 ± 3 14 (11; 15)	0.03
In-hospital mortality	6 (3)	5 (3.8)	1 (1.5)	0.66	0 (0)	0 (0)	-
Atrioventricular block III	19 (9.5)	16 (12.1)	3 (4.5)	0.08	15 (9.6)	4 (11.4)	0.75
Mean gradient after operation, mmHg	8.5 ± 3.4 8 (6; 11)	8.5 ± 3.5 8.4 (5.9; 11)	8.4 ± 3.3 8 (6; 11)	0.91	8.4 ± 3.3 8 (6; 11)	8.4 ± 3.6 8 (5.7; 11)	0.99
Aortic valve insufficiency							
0 1 2	171 (87.7) 22 (11.3) 2 (1.03%)	111 (86.7) 16 (12.5) 2 (1.3)	171 (87.7) 22 (11.3) 2 (1)	0.7	138 (88.5) 16 (10.3) 2 (1.3)	29 (82.9) 6 (17.1) 0 (0)	0.53
Paravalvular leak							
0 1 2	178 (91.3) 15 (7.7) 2 (1)	115 (89.8) 12 (9.4) 1 (0.8)	63 (94) 3 (4.5) 1 (1.5)	0.48	141 (90.4) 13 (8.3) 2 (1.3)	33 (94.3) 2 (5.7) 0 (0)	0.4
Stroke	7 (3.5)	6 (4.5)	1 (1.5)	0.42	6 (3.8)	0 (0)	0.59
Dialysis	11 (5.5)	9 (6.8)	2 (3)	0.34	6 (3.8)	0 (0)	0.59
ECMO use	3 (1.5)	3 (2.3)	0 (0)	0.55	2 (1.3)	0 (0)	1.0
Pacemaker after AVR	24 (12)	21 (15.8)	3 (4.5)	0.02	20 (12.7)	3 (8.6)	0.77
AICD	5 (2.5)	4 (3)	1 (1.5)	0.66	5 (3.2)	0 (0)	0.58
CRTD	1 (0.5)	1 (0.7)	0 (0)	1.0	0 (0)	1 (2.9)	0.18
Wound-healing disturbance	7 (3.5)	7 (5.3)	0 (0)	0.1	7 (4.5)	0 (0)	0.35

Data are expressed as mean ± standard deviation and median, interquartile range or numbers (percentages). AICD: automated implantable cardioverter defibrillator; AVR: aortic valve replacement; CRTD: *cardiac resynchronization therapy* with *defibrillator*; ECMO: extra-corporeal membrane oxygenation; LAHB: left anterior hemiblock; LBBB: left bundle branch block; RBBB: *right bundle branch block*.

**Table 4:** Follow-up at one year after deployment of EDWARDS INTUITY Elite rapid-deployment aortic valve.

	Total	New LBBB after AVR			Persistent New LBBB 1 Year after AVR		
		No (n = 133)	Yes (n = 67)	*p*-Value	No (n = 157)	Yes (n = 35)	*p*-Value
Mortality	8 (4)	7 (5.3)	1 (1.5)	0.27	-	-	-
Sinus rhythm	59 (30.7)	7 (5.6)	52 (78.8)	<0.001	29 (18.5)	30 (85.7)	<0.001
Atrial fibrillation	11 (5.7)	5 (4)	6 (9.1)	0.19	6 (3.8)	5 (14.3)	0.03
RBBB	8 (4.2)	7 (5.6)	1 (1.5)	0.26	8 (5.1)	0 (0)	0.35
LBBB	35 (18.2)	2 (1.6)	33 (50)	<0.001	0 (0)	35 (100)	<0.001
LAHB	6 (3.1)	3 (2.4)	3 (4.5)	0.41	6 (3.8)	0 (0)	0.59
Pacemaker	15 (7.8)	11 (8.7)	4 (6.1)	0.51	11 (7)	4 (11.4)	0.48
New pacemaker implantation during follow-up	1 (0.5)	0 (0)	1 (1.5)	0.34	0 (0)	1 (2.9)	0.18
AICD	1 (0.5)	0 (0)	1 (1.5)	0.34	1 (0.6)	0 (0)	1.0
CRTD	3 (1.6)	3 (2.4)	0 (0)	0.55	2 (1.3)	1 (2.9)	0.45

Data are expressed as mean ± standard deviation and median, interquartile range or numbers (percentages). AICD: automated implantable cardioverter defibrillator, AVR: aortric valve replacement, CRTD: *cardiac resynchronization therapy with defibrillator*, LAHB: left anterior hemiblock, LBBB: left bundle branch block, RBBB: *right bundle branch block*.

**Table 5:** Predictors of new LBBB directly after EDWARDS INTUITY Elite rapid-deployment aortic valve and persistent new LBBB after one year of follow-up period, univariate logistic regression analysis.

	OR	95% CI	*p*-Value
**New LBBB after aortic valve replacement**			
Euroscore II, %	0.834	0.720–0.936	<0.001
REDO operation, yes vs. no	0.164	0.026–0.584	0.003
Partial sternotomy, yes vs. no	2.137	1.151–3.978	0.016
PM after AVR, yes vs. no	0.250	0.057–0.761	0.012
Length of hospital stay, days	0.963	0.922–0.995	0.021
**Persistent new LBBB one year after aortic valve replacement**			
Body mass index, kg/m^2^	0.916	0.837–0.997	0.042
Euroscore II, %	0.831	0.669–0.972	0.015
REDO operation, yes vs. no	0.190	0.010–0.962	0.043
Length of operation, days	0.992	0.983–0.999	0.018
Length of hospital stay, days	0.905	0.820–0.973	0.002

AVR: aortic valve replacement, LBBB: left bundle branch block, PC: pacemaker.

**Table 6:** Predictors of new LBBB directly after EDWARDS INTUITY Elite rapid-deployment aortic valve and persistent new LBBB after one year of follow-up period, multivariate logistic regression analysis.

	OR	95% CI	*p*-Value
New LBBB after aortic valve replacement (no vs. yes)			
Euroscore II, %	1.202	1.063–1.404	0.001
Body mass index, kg/m^2^	1.067	0.995–1.148	0.068
PM implantation after AVR, yes vs. no	3.126	0.981–13.902	0.054
Persistent new LBBB one year after aortic valve replacement (yes vs. no)			
Atrial fibrillation, yes vs. no	17.622	3.029–152.422	0.001
The length of hospital stay, days	0.896	0.806–0.969	0.001
Body mass index, kg/m^2^	0.873	0.788–0.959	0.004
RBBB after AVR, yes vs. no	0.054	0.002–0.476	0.004

AVR: aortic valve replacement, LBBB: left bundle branch block, PM: pacemaker, RBBB: right bundle branch block.

## Data Availability

Data are available on a reasonable request.
